# An Information-Extreme Algorithm for Universal Nuclear Feature-Driven Automated Classification of Breast Cancer Cells

**DOI:** 10.3390/diagnostics15111389

**Published:** 2025-05-30

**Authors:** Taras Savchenko, Ruslana Lakhtaryna, Anastasiia Denysenko, Anatoliy Dovbysh, Sarah E. Coupland, Roman Moskalenko

**Affiliations:** 1 Department of Computer Science, Sumy State University, 40000 Sumy, Ukraine; 2 Department of Pathology, Sumy State University, 40000 Sumy, Ukraine; 3 Department of Eye and Vision Science, Institute of Life Course and Medical Sciences, University of Liverpool, Liverpool L69 7ZX, UK

**Keywords:** breast cancer, automated classification, information-extreme algorithm, cytological features, histopathology, whole-slide imaging, machine learning, digital pathology

## Abstract

**Background/Objectives:** Breast cancer diagnosis heavily relies on histopathological assessment, which is prone to subjectivity and inefficiency, especially with whole-slide imaging (WSI). This study addressed these limitations by developing an automated breast cancer cell classification algorithm using an information-extreme machine learning approach and universal cytological features, aiming for objective and generalized histopathological diagnosis. **Methods**: Digitized histological images were processed to identify hyperchromatic cells. A set of 21 cytological features (10 geometric and 11 textural), chosen for their potential universality across cancers, were extracted from individual cells. These features were then used to classify cells as normal or malignant using an information-extreme algorithm. This algorithm optimizes an information criterion within a binary Hamming space to achieve robust recognition with minimal input features. The architectural innovation lies in the application of this information-extreme approach to cytological feature analysis for cancer cell classification. **Results**: The algorithm’s functional efficiency was evaluated on a dataset of 176 labeled cell images, yielding promising results: an accuracy of 89%, a precision of 85%, a recall of 84%, and an F1-score of 88%. These metrics demonstrate a balanced and effective model for automated breast cancer cell classification. **Conclusions**: The proposed information-extreme algorithm utilizing universal cytological features offers a potentially objective and computationally efficient alternative to traditional methods and may mitigate some limitations of deep learning in histopathological analysis. Future work will focus on validating the algorithm on larger datasets and exploring its applicability to other cancer types.

## 1. Introduction

Breast cancer is the leading cause of cancer death among women worldwide [1]. According to the Global Cancer Observatory (GCO), in 2022, 2.3 million new cases of breast cancer were diagnosed worldwide, with 666,000 deaths [2]. Regions with high incidence rates of breast cancer include Asia (42.9%), Europe (24.3%), and North America (13.3%) [3].

Despite significant advances in the diagnosis and treatment of breast cancer through the introduction of modern imaging techniques such as Digital Breast Tomosynthesis (DBT)/3D tomography; the use of phase analysis, which allows the detection of malignant changes even in the parenchyma of high-density breast tissue; and the tremendous development of pathomorphological verification, the morbidity and mortality caused by breast cancer continue to remain high worldwide [4,5]. Today, immunohistology and molecular pathology have been fully integrated into the routine practices of cellular pathology services, contributing to the development and implementation of personalized medicine. However, artificial intelligence software solutions are still waiting for their breakthrough in this area of healthcare [6].

In addition, transformational changes in pathology have occurred due to the introduction of whole-slide imaging (WSI) and digital pathology, allowing for greater flexibility than a traditional light microscope [7]. Digitized whole-slide images (WSIs) have also increased the ability to study nuclear morphology in numerous histological specimens. However, the large sizes of WSIs—up to billions of pixels, containing thousands of nuclei—make exhaustive manual annotation impossible; thus, studies rely on hand-selected subregions of interest (ROI) rather than entire slides [8]. This technological innovation allows for detailed digital tissue images, significantly improving diagnostic and analytic processes and revealing new possibilities for process automation using computer vision and artificial intelligence [9]. Thus, automated methods are needed to fully quantify nuclear characteristics in WSIs. Using deep learning (DL) and image recognition algorithms significantly improves the accuracy and speed of diagnosis [10]. Artificial neural networks trained on large datasets, such as The Cancer Genome Atlas (TCGA) and ImageNet, can detect patterns and anomalies that may be missed by the human eye. This improves the quality of healthcare services and reduces the workload of pathologists, allowing them to focus on more complex cases [11].

In practice, cellular mutations caused by the oncological process in the mammary gland have a limited range of development options, regardless of the type of tissue. This applies to changes in cell shape, structure, and chemical composition. When classifying tumors, assessment of these features is extremely important, as it is key to establishing the correct diagnosis. Conventional diagnosis of breast cancer widely utilizes the Nottingham semi-quantitative system for assessing the most common non-specific type of breast cancer, i.e., one that does not have specific signs of lobular, mucinous, or tubular cancer or has mixed signs [12]. However, such assessment methods are difficult to implement using machine learning (ML) since they require some subjectivity in interpreting pathological images, and this interpretation will be related to each doctor’s experience. The Nottingham system consists of difficult-to-interpret signs that can differ significantly depending on the quality of the images. To overcome this scientific and methodological problem, pathology could be diagnosed using less ambiguous and well-differentiated signs. In particular, at the cellular level, the process of cell deformation occurs in a similar way in every tissue during neoplastic development.

This study aimed to develop an automated algorithm for classification of breast cancer cells based on universal nuclear features, thereby enhancing the objectivity and generalizability of histopathological diagnosis.

## 2. Materials and Methods

### 2.1. Tissue Sample Preparation and Ethics

This study was approved by the ethics committee of the Medical Institute of Sumy State University (Proceedings 3/12, 8 December 2022). Surgical tissue samples were provided by the Sumy Regional Clinical Oncology Hospital and the private clinic ‘MRIYA’ (Sumy, Ukraine), with informed consent secured from all patients prior to their participation. We obtained images of breast tumor nuclei from micropreparations that were prepared at the Scientific Center for Pathomorphological Research of the Department of Pathological Anatomy of Sumy State University. This study included 30 cases of invasive breast cancer of no special type (NST) (group 1) and 16 cases of benign breast tumors (BBTs) as a control group (group 2).

### 2.2. Histology

The tissue samples were fixed in 4% neutral buffered formaldehyde for 24 h, followed by dehydration, paraffin embedding, and sectioning at 4 μm using a Shandon Finesse 325 rotary microtome (Thermo Scientific, Waltham, MA, USA). The sections were then deparaffinized, dehydrated (using xylene and ethanol), and stained with hematoxylin and eosin. Digital images were acquired using a Zeiss Primo Star microscope equipped with a Zeiss Axiocam ERc 5s digital camera and the Zen 2.0 software package (Carl Zeiss, Jena, Germany).

### 2.3. Statistics

The normality of the data distribution was assessed using the Shapiro–Wilk test. For features exhibiting a Gaussian distribution, differences between groups were evaluated by two-tailed Student’s *t*-tests; nonparametric datasets were compared using the Mann–Whitney U test. Statistical significance was accepted at *p* < 0.05. All analyses were performed in Microsoft Office Excel 2016 with the AtteStat add-in (version 12.0.5).

To quantify classification performance, we computed the following standard metrics:

Accuracy measures the overall proportion of correctly classified cell instances—both malignant and normal—out of all predictions. It provides a general sense of model correctness but can be misleading in imbalanced datasets if one class dominates.

$$Accuracy=\frac{TP+TN}{TP+TN+FP+FN}$$

where TP (true positives) indicates the number of cells that are actually malignant (ground-truth label) that the classifier classifies as malignant; TN (true negatives) indicates the number of cells that are actually normal that the classifier correctly classifies as normal; FP (false positives) indicates the number of cells that are actually normal but the classifier incorrectly classifies as malignant (type I error); and FN (false negatives) indicates the number of cells that are actually malignant but the classifier fails to identify, instead classifying them as normal (type II error).

Precision quantifies the fraction of predicted malignant cells that are truly malignant. High precision indicates a low rate of false alarms (false positives), which is critical when the cost of mislabeling healthy cells as cancerous is high.

$$Precision=\frac{TP}{TP+FP}$$

Recall reflects the ability of the algorithm to detect malignant cells among all actual malignancies. A high recall minimizes missed cancerous cells (false negatives), an essential property in diagnostic contexts where overlooking a tumor can have serious consequences.

$$Recall=\frac{TP}{TP+FN}$$

The F_1_-score is the harmonic mean of precision and recall, balancing the trade-off between false positives and false negatives. It is especially informative when class distributions are imbalanced, summarizing a model’s ability to both identify malignancies and avoid false alarms.

$$F_{1}=2\;*\;\frac{Precision\;*\;Recall}{Precision+Recall}$$

All metrics were calculated using an independent test set of labeled cell images to ensure an unbiased evaluation of the classifier’s diagnostic accuracy and reliability.

## 3. Results

### 3.1. Analysis of the Subject Area and Data Collection

**Figure 1:**
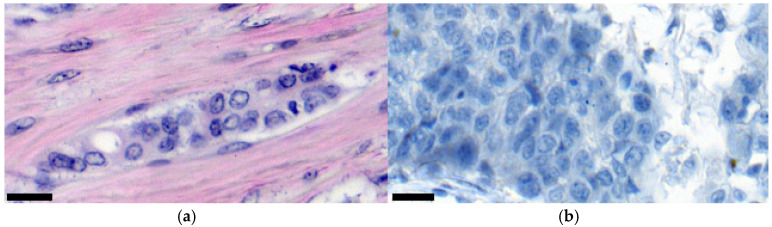
An example detailing breast cancer cells obtained under different conditions (**a**,**b**). Staining with hematoxylin–eosin. The magnification of the samples is indicated by markers in the lower left corners of the images (20 μm).

Automated analysis of histological images is complicated, first of all, by the mitotic activity of the affected cells and arbitrary conditions. For example, Figure 1a shows a tissue sample where visually distinguishing an oncological focus from healthy connective tissue is very easy. When the contours of the cells are precise, their nuclei and nucleoli can also be seen, allowing us to suspect a malignant tumor due to nuclear polymorphism.

In contrast, Figure 1b demonstrates a screening result where all available space is filled with tumor cells. Although their contours can still be separated visually, they are two completely different images with an ill-defined intersection of features. Of course, the contours of the affected cells can be distinguished in each of the images (Figure 1). Nevertheless, if we evaluate only cytological features, this is easier to perform in Figure 1a due to the clearer separation of the affected tissue from the connective tissue. On the other hand, Figure 1b clearly indicates the presence of progressive cancer; however, due to active proliferation, the tumor cells occupy the entire image. In turn, this complicates the diagnosis since the pathologist, based on his experience and knowledge, understands that this process is unlikely to cover the entire biopsy and that somewhere outside the image in Figure 1b there are areas of connective tissue. However, the algorithm, in its analysis, will rely only on the obtained image (Figure 1). Situations with type 1 statistical error are possible when the machine finds features that are actually not in an image, for example, connective tissue in Figure 1b.

First, when building the mitotic activity classifier it was worth making a technical restriction: a fixed scale for the input images. This was set to 400× magnification, as in Figure 1. In this way, we limited the recognition space, which allowed us to form three hypotheses regarding mitosis in a tissue:

1. Absence of affected cells. This is observed in cases where an image shows only healthy tissue.
2. Pronounced mitotic growth process. This means that most of the tissue in an image, at 400× magnification, is filled with a malignant neoplasm (>70%). From the point of view of diagnosis, this system can inform a doctor about the active development of oncology, where cell mitosis turns into active invasion. From images that fall into this class, it is possible to programmatically isolate the contours of affected cells since it is assumed that there are many of them in these images. In turn, this provides more information for further cytological analysis.
3. The beginning of the mitotic process/invasive growth. In general, these are situations where tumor cells and complexes are present in an image, but not many of them. At least at a magnification of 400×, healthy tissue should be visible. Also, these cases require additional analysis to detail tumorigenesis. Is this the initial stage of creating a cancer focus or the invasion of individual cells into the connective tissue?

Such a distinction allows one to reduce uncertainty at the stage of object classification. Setting an explicit limitation of the possible recognition options, each with specific characteristics, simplifies classification decisions. This facilitates identifying the object of interest, increasing the accuracy and interpretability of the result.

### 3.2. Nuclear Features of Breast Cancer

In making classification decisions, the primary metric was cytological (nuclear) features. In this case, each cell was described as a mathematical object consisting of 21 features conventionally divided into “shape” and “texture”. The first cluster characterized geometric properties, and the second characterized visual ones (Table 1).

**Figure 2:**
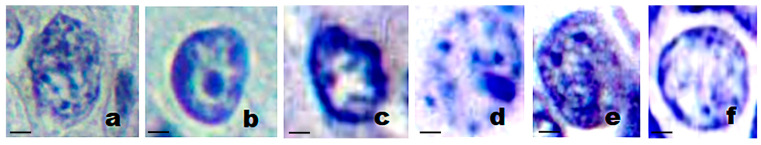
Examples of images from the training dataset. Different variants of chromatin distribution in the nucleus (**a**–**f**) indicate and help identify atypical cells.

To form the training dataset, 167 cell images were taken at a magnification of 400×. Of these, 124 images represented cells that characterized pathological tissue processes, and 43 images represented normal tissues. The discrepancy in these numbers was justified by the fact that the signs of healthy cells are very uniform, so 43 images were enough to describe their main cytological parameters. Figure 2 shows examples of cell images that were used during this study.

By encoding each cell in terms of a concise set of cytological features, we leveraged the fact that pathological transformations follow a limited repertoire of morphologies, regardless of tissue origin. This constraint enabled us to reduce the classifier’s label space and focus exclusively on the mutation-driven patterns most characteristic of oncogenic processes.

Nuclear enlargement, irregular contours, and altered chromatin organization arise from genomic instability, aberrant mitotic activity, and dysregulated gene expression in cancer cells. By quantifying these features objectively, we captured reproducible markers of malignancy that transcended the observer bias inherent to manual grading systems. Moreover, because cytological alterations follow a limited repertoire of pathological morphologies, they provided a concise yet powerful feature space for machine learning. In this study, leveraging standardized measurements of area, shape, and texture enabled sensitive discrimination between healthy and malignant cells; facilitated the development of generalizable decision rules; and supported rapid, high-throughput analysis of digitized histological specimens. We conducted an initial assessment of the dataset using some of these features.

The nuclear area (*A*) quantifies the two-dimensional size of each cell nucleus in pixel units. Changes in *A* are indicative of nuclear enlargement, a hallmark of many neoplastic processes. Consistently larger *A* values in malignant populations reflect dysregulated growth and increased DNA content.

$$A=\sum_{(x,y)\in \Omega }1$$

where Ω is the set of all pixels belonging to the segmented nuclear mask.

The *N*/*C* ratio captures the relative enlargement of the nucleus compared to the surrounding cytoplasm. Elevated *N*/*C* ratios are characteristic of oncogenic transformation, reflecting increased nuclear content and reduced cytoplasmic volume. This metric is widely used in cytopathology to distinguish high-grade malignancies.

$$\frac{N}{C}ratio=\frac{A_{nucleus}}{A_{cell}-A_{nucleus}}$$

where $A_{cell}$ is the area of the entire cell (nucleus + cytoplasm).

Circularity (*C*) measures how closely the nuclear outline approximates a perfect circle (*C* = 1). Deviations from unity indicate nuclear membrane irregularities and pleomorphism. Lower *C* values are associated with malignant cells, which often exhibit lobulated or indented nuclear contours.

$$C=\frac{4\pi A}{P^{2}}$$

where *A* is the nuclear area and *P* is the nuclear perimeter.

Chromatin entropy (*H*) quantifies the heterogeneity of pixel intensities within the nucleus, reflecting chromatin organization and staining variability. Higher *H* values indicate greater textural complexity and irregular chromatin patterning, which are commonly observed in malignant nuclei due to uneven chromatin condensation and distribution.

$$H=-\sum_{i=0}^{L-1}p_{i}\log_{2}\left(p_{i}\right)$$

where $p_{i}$ is the normalized frequency of gray level *i* within the nuclear region and *L* is the number of intensity levels.

As illustrated in Figure 2, cells exhibiting malignant cytology showed a mean nuclear area of 3730 ± 280 px^2^ versus 2093 ± 60 px^2^ for normal cells (Table 2). Malignant nuclei also presented significantly reduced circularity (0.68 ± 0.04 vs. 0.89 ± 0.02) and elevated chromatin entropy (4.31 ± 0.23 vs. 2.86 ± 0.06). These pronounced disparities underscore the discriminative power of our selected cytological features.

### 3.3. Analysis of Oncological Image Recognition Methods

Early machine learning methods for breast cancer cytology often relied on hand-crafted features extracted from fine-needle aspiration (FNA) slides. For example, an eXtreme Gradient Boosting (XGB) classifier built on first-order intensity and shape descriptors achieved accuracies around 92% on FNA datasets of comparable size (*n* ≈ 200), demonstrating the value of textural and geometric metrics in malignancy detection [13]. However, tree-based ensembles can overfit when the feature space is large relative to the sample count and generally lack an explicit information-theoretic decision criterion.

In recent years, convolutional neural networks (CNNs) applied directly to histopathological patches have pushed accuracy above 94%. A CNN-based pipeline for invasive cell classification reported average accuracy and F1-score values of 95% and 89% in multi-center cohorts [14]. Meanwhile, hybrid meta-learning frameworks combining multiple model outputs via meta-classifiers have achieved accuracy and F1-score values of up to 98.7% and 98%, albeit with substantially larger training sets and complex ensembling strategies [15]. These deep and meta-learning solutions excel in end-to-end feature learning but require extensive computational resources and risk brittleness to image-acquisition variability.

While modern deep learning pipelines for breast cancer histology routinely tout accuracies in the mid-90% range, they do so by training on thousands—even tens of thousands—of image patches and by leaning on complex network architectures that can feel like “black boxes”. In contrast, our information-extreme method delivers nearly 90% accuracy based on under 200 hand-labeled cell images. Instead of millions of weights, it relies on 21 well-understood cytological features, including nuclear size, shape, and texture, and optimizes a clear, information-theoretic criterion to carve out decision boundaries in a binary Hamming space.

This lean feature-driven strategy offers two key advantages. First, it remains interpretable: every classification boils down to how far a cell’s measured properties lie from the hyperspherical “containers” we have tuned, making it easy to trace why a nucleus was flagged as malignant. Second, it is computationally lightweight with no need for GPUs or lengthy back-propagation, so the entire pipeline runs comfortably on a standard lab workstation.

### 3.4. Recognition Method

The information-extreme algorithm was chosen as the primary machine learning method, as it allows unifying decision rules by transferring them to the binary Hamming space. It is assumed that in this way, the system is better able to recognize the least pronounced features or those at the intersection of several recognition classes with a minimal mathematical description of an object. The main idea of the information-extreme algorithm is to search for the global maximum of the information criterion averaged over the alphabet of recognition classes in the working (allowable) domain of its function definition:

(1) $$\delta_{K}^{*}=\arg\{\underset{G_{\delta }}{\max}\left\{\underset{G_{E}\cap \{k\}}{max}{\bar{E}}^{\left(k\right)}\right\}\}$$

where $\delta_{K}^{*}$ is the optimal parameter of the control tolerance field; $G_{\delta }$ is the permissible range of values of parameter δ of the control tolerance field; $\left\{k\right\}$ is a time-ordered set of machine learning steps; and $G_{E\;}$ is the permissible domain of the definition of the information criterion function.

The input for a machine learning algorithm is an array $\left\{y_{m,i}^{\left(j\right)}\right\}$, a system of normalized tolerance fields $\left\{\delta_{H,i}\right\}$ on recognition features, which specifies the range of values of the control tolerance system.

As a criterion for optimizing the parameters of Decision Support System (DSS) machine learning, the modified Kullback information measure was used in the following form:

(2) $$E_{m}^{\left(k\right)}=\frac{1}{n_{min}}{log}_{2}\left\{\frac{2n_{min}+10^{-r}-\left[K_{1}^{\left(k\right)}+K_{2}^{\left(k\right)}\right]}{\left[K_{1}^{\left(k\right)}+K_{2}^{\left(k\right)}\right]+10^{-r}}\right\}|n_{min}-(K_{2}^{\left(k\right)}-K_{3}^{\left(k\right)})|$$

The normalized modification of criterion (2) had the following form (3):

(3) $$E_{m}^{\left(k\right)}=\frac{E_{K_{m}}^{\left(k\right)}}{E_{K_{max}}^{\left(k\right)}}$$

where $K_{1}^{\left(k\right)}$ is the number of events in which the feature vectors of recognition class $X_{h,s,m}^{o}$ are mistakenly not assigned to it; $K_{2}^{\left(k\right)}$ is the number of events in which the feature vectors of the neighboring recognition class are mistakenly assigned to recognition class $X_{m}^{o}$; $n_{min}$ is the minimum size of the representative training sample; and $10^{-r}$ is a sufficiently small number that is entered to avoid division by zero.

Based on the obtained optimal geometric parameters of the recognition class containers, production decision rules were constructed, which had the following form:

(4) $$\left(\forall X_{m}^{o}\in R^{\left|M\right|}\right)\left(\forall x^{\left(j\right)}\in R^{\left|M\right|}\right)\left[\begin{array}{c} if\left(\mu_{m}>0\right)\left(\mu_{m}=\max\left\{\mu_{m}\right\}\right)thenx^{\left(j\right)}\\ \in X_{m}^{o}elsex^{\left(j\right)}\notin X_{m}^{o} \end{array}\right]$$

where $x^{\left(j\right)}$ is the recognized vector and $\mu_{m}$ is the function of belonging of vector $x^{\left(j\right)}$ for the container of recognition class $X_{m}^{o}.$

In expression (4), the membership function for the hyperspherical container of recognition class $X_{m}^{o}$ is defined by Formula (5):

(5) $$\mu_{m}=1-\frac{d(x_{m}^{*}⊕x^{\left(j\right)})}{d_{m}^{*}}$$

where $x_{m}^{*}$ and $d_{m}^{*}$ are optimal machine learning parameters: the averaged binary implementation and radius of the hyperspherical container, respectively.

Thus, at the stage of object classification, according to the decision rules (5), it is determined whether a recognized cell belongs to one of the classes given by the alphabet $X_{m}^{o}$.

**Figure 3:**
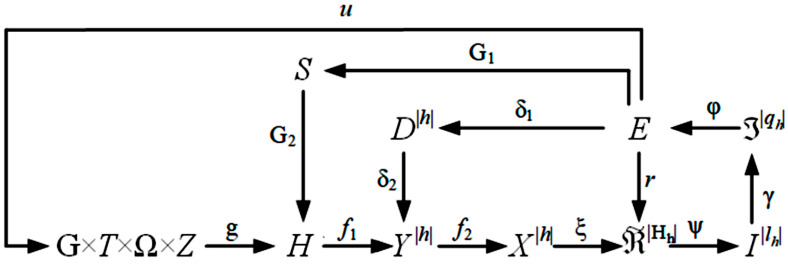
Functional categorical model of machine learning.

Thus, the functional categorical model of an information-extreme machine learning system for recognizing breast cancer will have the form provided in Figure 3.

In Figure 3, cartesian product T × G × Ω × Z specifies the source of information. The term set (E) of the values of the information criterion (3) is common to all optimization contours of the machine learning parameters. Operator r: $E\to R^{\left|M_{h}\right|}$ is restored for each stratum of the decursive tree in the process of information-extreme machine learning, in the general case, a fuzzy partition ($R^{\left|M_{h}\right|}$) of recognition classes. Operator ξ maps the binary vectors of the working matrix (X^|h|) to the partition ($R^{\left|M_{h}\right|}$). Next, operator $\psi :X_{h}\to I^{\left|I_{h}\right|}$ tests the main statistical hypothesis ($\gamma_{1}:x_{h,n}^{j}\;\in X_{h,m}^{o}$). Operator γ calculates the set of precision characteristics ($I^{\left|q_{h}\right|}$) where $q_{h}=l_{h}$. At each step of machine learning, operator φ calculates the information criterion of optimization, which is a function of the precision characteristics. The cycle of optimization of the control tolerances is closed through the set $D^{\left|h\right|}$ whose elements are the values of the control tolerances for diagnostic features. Operator $u_{H}$ regulates the machine learning process.

### 3.5. The Workflow of the Image Recognition Algorithm

All histological sections (4 μm) were stained with hematoxylin and eosin and digitized at 400× magnification on a Zeiss Primo Star microscope with an Axiocam ERc 5s camera. Raw TIFFs of whole-slide images or manually selected regions of interest (ROI) were exported under uniform illumination settings. Preprocessing began with color deconvolution to isolate the hematoxylin channel, followed by global thresholding (mean + 2 σ) to detect hyperchromatic pixels. Morphological opening/closing and connected component analysis then defined candidate ROIs, excluding objects < 500 px^2^ and those touching image borders. Within each ROI, nuclei segmentation employed adaptive thresholding, seeded watershed splitting to resolve clusters, and active contour refinement, with masks filtered by solidity (≥0.6) and eccentricity (≤0.98) and manually reviewed as needed. From each binary mask, we extracted 21 cytological features—10 geometric descriptors (e.g., area, perimeter, circularity, axis lengths, and solidity) and 11 texture metrics (first-order statistics and gray-level co-occurrence measures such as contrast, ASM, energy, and correlation)—all computed in pixel units. These descriptors were concatenated into per-cell feature vectors, and each dimension was tested for normality (Shapiro–Wilk test) and normalized via z score or rank transformation. Classification was performed in a binary Hamming space using an information-extreme algorithm: hyperspherical container radii and tolerance field parameters were optimized by maximizing a modified Kullback–Leibler criterion (Equations (1)–(3)), and membership functions assigned each vector to “normal” or “malignant”. Finally, predicted labels were overlaid on the original ROI to produce annotated maps and structured pathology reports for downstream analysis.

**Figure 4:**
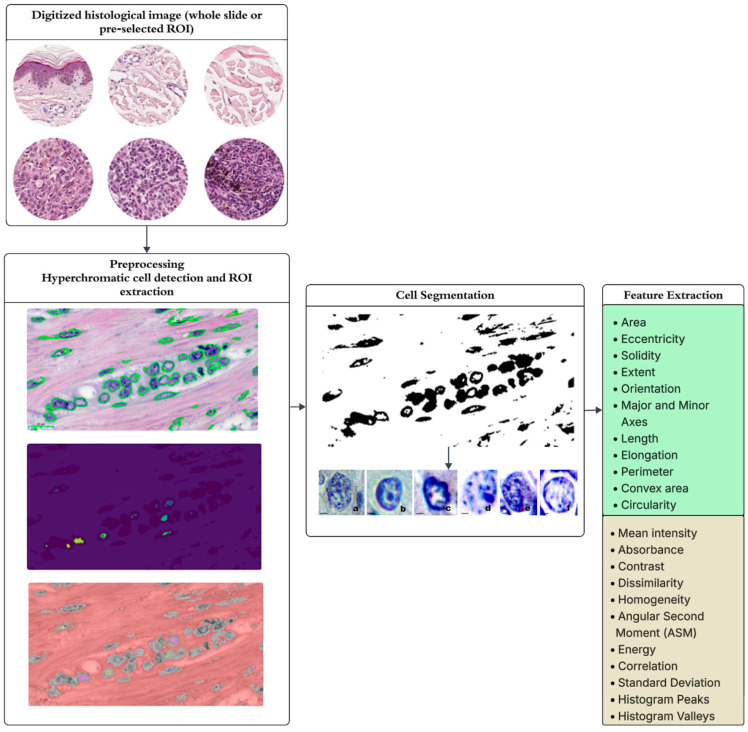
The workflow of the image feature Extraction algorithm.

**Figure 5:**
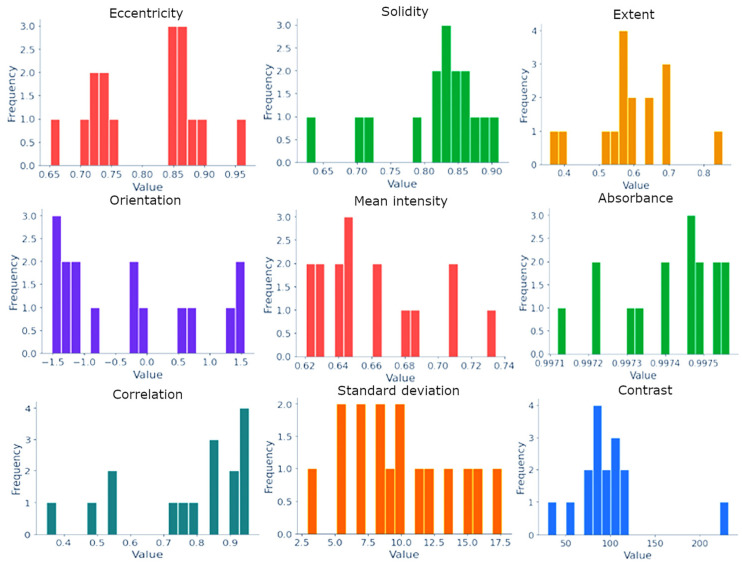
Nuclear features.

In the first step, the algorithm identifies the damaged tissue area by isolating cells with marked hyperchromia (Figure 4). Subsequently, classification of the area based on nuclear features (Table 1) is performed, with an example of feature distribution shown in Figure 5 through transformation into mathematical objects.

Consider the process of identifying breast cancer using a histological image (Figure 1a). The initial step involves determining the area of tissue damage by isolating cells exhibiting noticeable hyperchromia (Figure 4).

In this instance, the lesion occupies 17% of the biopsy, which is insufficient for diagnosis. Further classification of the region of interest based on nuclear features is necessary. Consequently, the system selected approximately 40 cells in Figure 4 and transformed them into mathematical objects for subsequent analysis by the intelligent component. Some of their nuclear features are illustrated in Figure 5: Eccentricity measures the ratio of the distance between the foci of a cell to the length of its major axis. The closer this indicator is to one, the more elliptical the cell shape, which approaches a straight line. Solidity is the ratio of the area of a cell to its perimeter. A low value may indicate an abnormal morphology or damage to the cell membrane. The extent is the ratio of the area of a cell to the area of its minimum describing rectangle. It indicates how compact an object is, and a low value indicates an abnormal cell shape. The orientation is the angle at which an object is directed. It can help understand the alignment and, possibly, the dynamics of cellular structures. The mean intensity is the average pixel intensity in a cell region, which can help determine the structural and chemical properties of a tissue. Absorbance measures the amount of light absorbed by a sample. In microscopy or medical imaging, absorbance can sometimes be measured by how much light is blocked or absorbed by an image region, which is often related to the density or thickness of the sample. Correlation determines how linearly dependent pixels are on each other in a texture matrix. High correlation may indicate structural integration and orderliness in a tissue. The Standard Deviation assesses the diversity of pixel intensity values in an area of interest. A high Standard Deviation may indicate tissue heterogeneity, which is typical of many pathologies. Contrast is a measure of differences in intensity between adjacent pixels. High contrast in a histological image may indicate significant structural changes in the tissue, such as fibrosis or proliferation.

Primary attention was drawn to the high concentration of cells with an extent index < 0.7, indicating a transient process of cellular mutation and gradual degradation of their shape, along with high absorbance values suggestive of pronounced hyperchromia in the area of interest (Figure 4). Concurrently, the solidity index suggested an early stage of the process, as cell membranes remained intact. The mean intensity indicated color similarity within the regions of interest, suggesting the presence of pathology.

The intelligent component performed further analysis and detected hidden interactions between nuclear features, receiving a mathematical description of the cells as a 21-feature vector (10 geometric features and 11 textural features), enabling a more comprehensive assessment of the tissue and lesions.

**Figure 6:**
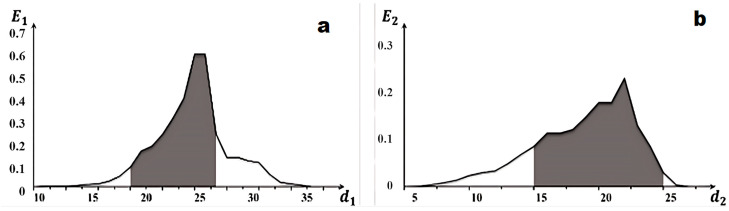
Graphs of the dependence of criterion (3) on the radii of the recognition class containers for (**a**) a normal cell ($X_{1}^{o}$) and (**b**) a malignant cell ($X_{2}^{o}$)).

Decision rules were constructed following the acquisition of optimal machine learning parameters in terms of information content [16]. Their geometric parameters are presented in graphs illustrating the dependence of the information criterion on the radii of the recognition class containers (Figure 6).

According to the presented graphs, the optimal radii of the recognition class containers are r_normal_ = 25 (here and hereafter in code units) for the normal cell class and r_malignant_ = 21 for the malignant cell class.

Consequently, 16 cells were classified as malignant. Although the initial segmentation of the image (Figure 2) identified approximately 40 areas suspected of being oncological, the majority of the cells did not fall into the malignant class because they lacked sufficient nuclear features for verification by the intelligent component.

**Figure 7:**
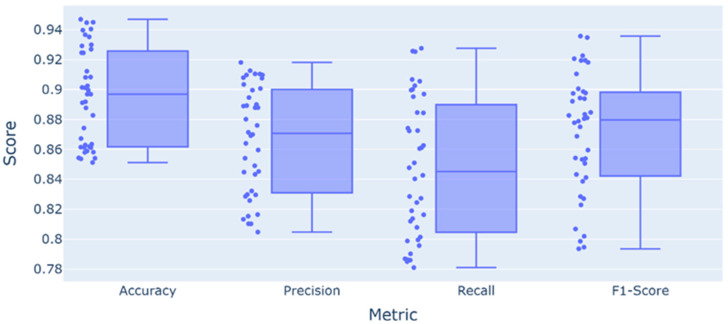
Distribution of performance metrics.

To evaluate the functional efficiency of the intelligent model for recognizing cell images, a dataset of 176 labeled cell images was created. The algorithm’s performance was assessed by calculating four recognition system parameters (Figure 7): accuracy, precision, recall, and the F1-score. Accuracy reflects the proportion of correctly classified cases. Precision indicates the rate of false positives for the malignant cell class. Recall reflects the system’s ability to identify malignant cells. The F1-score is the harmonic mean of precision and recall. In Figure 6, the accuracy is 89%, the precision is 85%, and the recall is 84%. The F1-score is 88%, indicating a balanced model that correctly identifies malignant cells without a significant number of errors.

Precision and recall are the primary metrics for algorithm tuning, as they provide a more comprehensive understanding of the cancer diagnosis task. A model with high accuracy but low recall would miss malignant cells more frequently. Imbalance is also possible in models with high precision and low recall, where benign cells would be misclassified as malignant more often. In a clinical context, the cost of false negatives (missing a malignant cell) is typically higher than that of false positives. Therefore, optimizing for better recall, even with a slight reduction in precision, may be a preferable strategy. The F1-score serves as a guideline to ensure the overall reliability of the model’s performance. It is also important to note that the system operates on histological images at 400× magnification. This implies that even a relatively small number of cells classified as malignant may indicate an active stage of mitosis with gradual invasion into the connective tissue. Furthermore, this scale imposes additional scientific and methodological limitations on the classifier, particularly significant overlap in recognition features. As most presented classes will exhibit similar visual characteristics, expanding the feature space will be essential, for instance, by including an assessment of surrounding tissues or the inter-center distances between cells to identify conglomerates. 

## 4. Discussion

Traditionally, pathomorphological diagnosis of breast cancer has relied heavily on visual assessment of cellular and nuclear morphological features, such as size, shape, the nuclear–cytoplasmic ratio, and the chromatin status [16,17]. While these methods are fundamental, their subjectivity and dependence on the pathologist’s experience can lead to inter- and intra-observer variability. Furthermore, quantitative evaluation of these features, especially when analyzing large volumes of whole-slide imaging (WSI) data, is a laborious and inefficient process [18].

The automated breast cancer cell classification algorithm proposed in this study, based on an information-extreme approach and analysis of universal cytological features, aims to overcome these limitations [19]. In contrast to manual or semi-automated methods [20], our approach provides an objective, quantitative assessment of cellular morphological characteristics, reducing the influence of subjective interpretation.

Existing machine learning algorithms, including deep learning methods, have demonstrated significant potential in the classification of breast cancer histopathological images. Architectures like convolutional neural networks (CNNs) and, more recently, Transformers have shown remarkable success in various medical image analysis tasks [21,22,23]. These advanced architectures often incorporate mechanisms like attention blocks [24] to enhance feature extraction and focus on salient regions within images. While these deep learning approaches have achieved high performance, they can also suffer from over- or underestimation of certain tumor types and may be sensitive to variability in sample preparation and image quality [25].

Our information-extreme algorithm utilizing a set of universal nuclear features offers an alternative approach. Instead of relying on complex deep learning architectures, it focuses on robust classification of cells based on a carefully selected set of interpretable cytological characteristics. This design choice prioritizes generalizability and may have lower computational requirements compared to deep learning models.

A key distinction of our approach is its emphasis on universal cytological features that reflect fundamental changes that occur in cells during the neoplastic process, regardless of the histological type of the tumor [26]. This contrasts with methods that may focus on specific morphological characteristics of certain cancer subtypes, which can limit their applicability. The use of less ambiguous and well-differentiated features at the cellular level, such as changes in shape, structure, and chemical composition, makes our approach more suitable for automated analysis compared to semi-quantitative systems like the Nottingham system [27], which rely on more subjective criteria.

The obtained results (89% accuracy, 85% precision, 84% recall, and an F1-score of 88%) demonstrate the high efficiency of the developed algorithm in classifying breast cancer cells based on the selected cytological features. These indicators are competitive with the results of other studies in this field [28].

Of course, our method does not aim to replace deep learning systems where massive datasets and end-to-end image learning are available. Rather, it sits in a sweet spot for smaller labs or pilot studies: you obtain a transparent algorithm that is quick to train, simple to deploy, and still highly effective at teasing apart healthy and cancerous cells.

Future research directions will include expanding the set of analyzed cytological features, particularly investigating spatial relationships between cells and characteristics of the surrounding tissue to further improve classification accuracy and the ability to detect more complex pathological changes. Furthermore, we plan to validate the developed algorithm on independent datasets and compare it with other state-of-the-art classification algorithms, not only in the context of breast cancer but also for other types of oncological diseases where universal cytological features can be applied.

## 5. Conclusions

In this study, an automated breast cancer cell classification algorithm based on an information-extreme approach and analysis of universal cytological features was developed and tested. The proposed method demonstrated high efficiency in classifying malignant cells, achieving an accuracy of 89%, a precision of 85%, a recall of 84%, and an F1-score of 88% on the created dataset.

The obtained results indicate the potential of the developed algorithm as an objective and quantitative tool for analysis of breast cancer histopathological images. The use of universal cytological features reflecting fundamental changes in cells during the neoplastic process ensures less dependence on subjective pathologist interpretation and the specific characteristics of individual cancer subtypes. This makes the proposed approach promising for the creation of more generalized and robust automated diagnostic systems.

In contrast to laborious manual and semi-automated methods, as well as the potential limitations of deep learning algorithms regarding image variability and over- or underestimation of certain tumor types, the developed information-extreme algorithm offers an efficient way to quantitatively assess cellular morphological features.

Future research will focus on expanding the set of analyzed cytological features, including spatial characteristics and features of the surrounding tissue, as well as validating the algorithm on independent datasets and comparing it with other state-of-the-art classification methods for various types of oncological diseases. This will allow an evaluation of the universality and scalability of the proposed approach for broader application in clinical practice.

## Figures and Tables

**Table 1:** Nuclear features.

Type	Name	Description
**Shape**	Area	It may indicate the stage of cancer development and malignant cell growth. In most cases, a large area is characteristic of tumorous or hyperactive cells.
	Eccentricity	Measures the ratio of the distance between the foci of a cell to the length of its major axis. The closer this indicator is to one, the more elliptical the cell shape, which approaches a straight line.
	Solidity	The ratio of the area of a cell to its perimeter. A low value may indicate an abnormal morphology or damage to the cell membrane.
	Extent	The ratio of the area of a cell to the area of its minimum describing rectangle. Indicates how compact an object is, and a low value indicates an abnormal cell shape.
	Orientation	The angle at which an object is directed. Can help understand the alignment and, possibly, the dynamics of cellular structures.
	Major and Minor Axis Lengths	Measurements of the major and minor axis lengths. Their ratio can help identify anomalies in the shape of an object.
	Elongation	Quantifies how elongated the shape of an object is and is calculated as the ratio of the length of the major axis to the length of the minor axis.
	Perimeter	The length of a cell’s outline. In some cases, it may indicate non-uniformity.
	Convex area	The ratio of a cell’s area to its convex area, which indicates the degree of contour protrusion.
	Circularity	Measures how close a cell’s shape is to a perfect circle.
**Texture**	Mean intensity	The average pixel intensity in a cell region can help determine the structural and chemical properties of a tissue.
	Absorbance	Measures the amount of light absorbed by a sample. In microscopy or medical imaging, absorbance can sometimes be measured by how much light is blocked or absorbed by an image region, which is often related to the density or thickness of the sample.
	Contrast	A measure of the differences in intensity between adjacent pixels. High contrast in a histological image may indicate significant structural changes in a tissue, such as fibrosis or proliferation.
	Dissimilarity	Reflects the differences between pixel values in a texture matrix. High values may indicate significant tissue heterogeneity, which is often found in tumor processes.
	Homogeneity	Indicates how close the pixel values in a texture matrix are to each other. Higher uniformity may indicate less aggressive or less altered tissues.
	Angular Second Moment (ASM)	A measure of texture uniformity that measures the squares of the values in a texture matrix. Higher ASM values indicate greater regularity and less textural complexity.
	Energy	The square root of ASM also reflects texture uniformity. A high energy score indicates greater regularity and texture uniformity.
	Correlation	Determines how linearly dependent pixels are on each other in a texture matrix. High correlation may indicate structural integration and orderliness in a tissue.
	Standard Deviation	Assesses the diversity of pixel intensity values in an area of interest. A high Standard Deviation may indicate tissue heterogeneity, which is typical of many pathologies.
	Histogram Peaks	Peaks in an intensity histogram may indicate the dominant intensity levels in a sample, which helps identify specific cellular components or structures.
	Histogram Valleys	Low points between peaks in a histogram may indicate the presence of different cell types or structures in a tissue.

**Table 2:** Average cytological feature measurements for training dataset.

Class	Nucleus Area (px^2^)	*N*/*C* Ratio	Circularity	Chromatin Entropy
Pathological	3737.5	0.7075	0.6750	4.3875
Normal	2093.3	0.4833	0.8933	2.8567

## Data Availability

Data are available within this article.
